# FSHing for DNA Damage: Key Features of MutY Detection of 8-Oxoguanine:Adenine Mismatches

**DOI:** 10.1021/acs.accounts.3c00759

**Published:** 2024-03-12

**Authors:** Chandrima Majumdar, Merve Demir, Steven R. Merrill, Mohammad Hashemian, Sheila S. David

**Affiliations:** Department of Chemistry, University of California, Davis, California 95616, United States

## Abstract

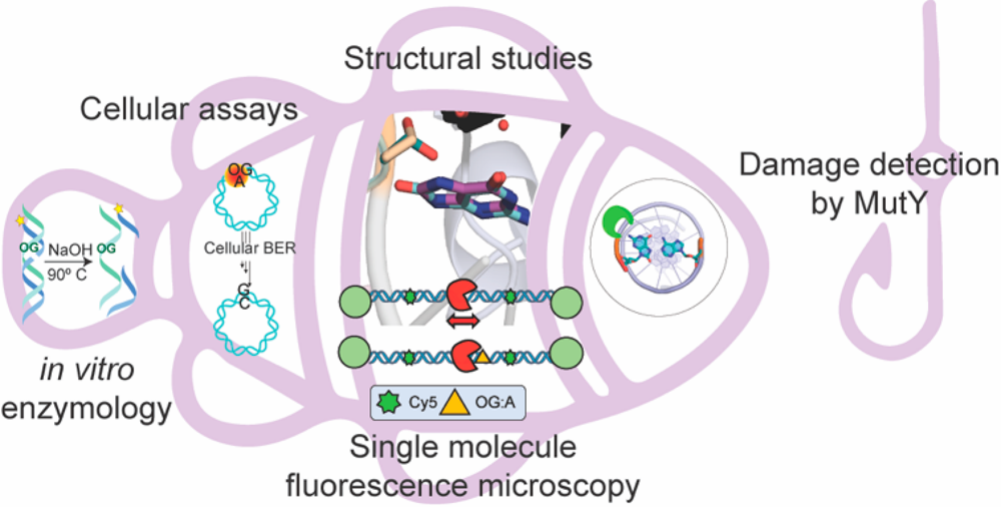

Base excision repair (BER) enzymes are genomic superheroes that stealthily and accurately identify and remove chemically modified DNA bases. DNA base modifications erode the informational content of DNA and underlie many disease phenotypes, most conspicuously, cancer. The “OG” of oxidative base damage, 8-oxo-7,8-dihydroguanine (OG), is particularly insidious due to its miscoding ability that leads to the formation of rare, pro-mutagenic OG:A mismatches. Thwarting mutagenesis relies on the capture of OG:A mismatches prior to DNA replication and removal of the mis-inserted adenine by MutY glycosylases to initiate BER. The threat of OG and the importance of its repair are underscored by the association between inherited dysfunctional variants of the MutY human homologue (MUTYH) and colorectal cancer, known as MUTYH-associated polyposis (MAP). Our functional studies of the two founder MUTYH variants revealed that both have compromised activity and a reduced affinity for OG:A mismatches. Indeed, these studies underscored the challenge of the recognition of OG:A mismatches that are only subtly structurally different than T:A base pairs. Since the original discovery of MAP, many MUTYH variants have been reported, with most considered to be “variants of uncertain significance.” To reveal features associated with damage recognition and adenine excision by MutY and MUTYH, we have developed a multipronged chemical biology approach combining enzyme kinetics, X-ray crystallography, single-molecule visualization, and cellular repair assays. In this review, we highlight recent work in our laboratory where we defined MutY structure–activity relationship (SAR) studies using synthetic analogs of OG and A in cellular and *in vitro* assays. Our studies revealed the 2-amino group of OG as the key distinguishing feature of OG:A mismatches. Indeed, the unique position of the 2-amino group in the major groove of OG_*syn*_:A_*anti*_ mismatches provides a means for its rapid detection among a large excess of highly abundant and structurally similar canonical base pairs. Furthermore, site-directed mutagenesis and structural analysis showed that a conserved C-terminal domain β-hairpin “FSH’’ loop is critical for OG recognition with the “His” serving as the lesion detector. Notably, MUTYH variants located within and near the FSH loop have been associated with different forms of cancer. Uncovering the role(s) of this loop in lesion recognition provided a detailed understanding of the search and repair process of MutY. Such insights are also useful to identify mutational hotspots and pathogenic variants, which may improve the ability of physicians to diagnose the likelihood of disease onset and prognosis. The critical importance of the “FSH” loop in lesion detection suggests that it may serve as a unique locus for targeting probes or inhibitors of MutY/MUTYH to provide new chemical biology tools and avenues for therapeutic development.

## Key References

- ManloveA. H.; McKibbinP. L.; DoyleE. L.; MajumdarC.; HammM. L.; DavidS. S.Structure-Activity Relationships Reveal Key Features of 8-Oxoguanine: A Mismatch Detection by the MutY Glycosylase. ACS Chem. Biol.2017, 12, 2335–234410.1021/acschembio.7b0038928723094 PMC5603899.^1^*In this work, we compared the impact of OG substrate modifications on kinetic parameters of base excision, mismatch affinity, and lesion repair in cells. The results showed the heavy reliance on the detection and verification of OG for efficient repair activity of MutY.*
- MajumdarC.; McKibbinP. L.; KrajewskiA. E.; ManloveA. H.; DavidS. S.Unique H-Bonding Pattern of Adenine with the Oxidatively Damaged Base 8-Oxoguanine Enables Specific Recognition and Repair by DNA Glycosylase MutY. J. Am. Chem. Soc.2020, 142, 20340–2035010.1021/jacs.0c0676733202125 PMC9187209.^2^*MutY structure–activity relationships with adenine-analog substrates revealed that the feature most strongly required for efficient repair is the ability of the A analog to base pair with the syn conformer of OG, rather than intrinsic nucleobase lability.*
- RusselburgL. P.; O’Shea MurrayV. L.; DemirM.; KnutsenK. R.; SehgalS. L.; CaoS.; DavidS. S.; HorvathM. P.Structural Basis for Finding OG Lesions and Avoiding Undamaged G by the DNA Glycosylase MutY. ACS Chem. Biol.2020, 15, 93–10210.1021/acschembio.9b0063931829624 PMC7069122.^3^*Structural studies along with site-directed mutagenesis revealed the role of the conserved C-terminal domain β-hairpin “FSH’’ loop of MutY in recognition of OG over G.*
- LeeA. J.; MajumdarC.; KatheS. D.; Van OstrandR. P.; VickeryH. R.; AverillA. M.; NelsonS. R.; ManloveA. H.; McCordM. A.; DavidS. S.Detection of OG:A Lesion Mispairs by MutY Relies on a Single His Residue and the 2-Amino Group of 8-Oxoguanine. J. Am. Chem. Soc.2020, 142, 13283–1328710.1021/jacs.0c0428432664726 PMC7593828.^4^*Single-molecule and ensemble assays, along with cellular repair, illuminated the role of a single His within the FSH loop in the detection OG_syn_:A_anti_ base pairs via the unique major groove position of the 2-amino of OG.*

## Introduction: The “OG” of DNA Base Lesions and BER “GO” to the Rescue

The free radical theory of aging posits that aging is a result of the cumulative damage inflicted upon cells by reactive oxygen and nitrogen species (RONS).^5^ RONS, arising from environmental sources, oxidative metabolism, or inflammation, react with cellular macromolecules compromising structure and function.^6,7^ DNA base and sugar modifications resulting from RONS, exacerbated by faulty repair mechanisms, lead to myriad of consequences, such as transcriptional arrest and replication errors, ultimately leading to genomic instability, carcinogenesis, aging, and other disease phenotypes.^8−10^

**Figure 1:**
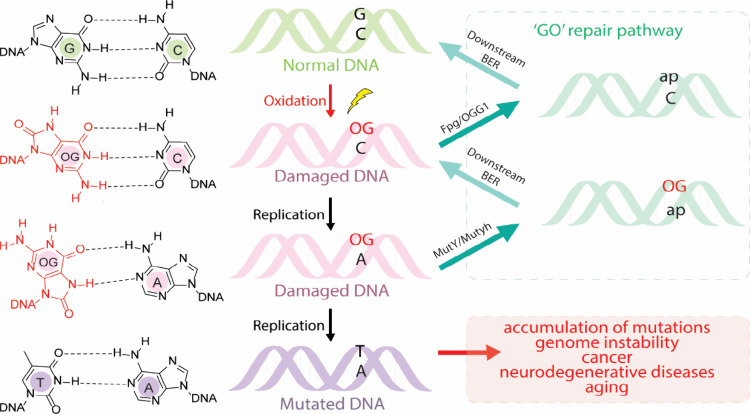
Presence of OG in DNA leads to the formation of G:C to T:A transversion mutations. The GO repair pathway features two base excision repair (BER) glycosylases, Fpg/OGG1 and MutY/MUTYH, that prevent mutations associated with OG by removing OG from OG:C base pairs or preventing propagation of the pro-mutagenic OG:A base pairs by removal of the misplaced A, respectively. The abasic site (designated as “ap”) product is removed and replaced using the intact strand as the guide by downstream BER pathway enzymes. Note that replicative polymerases more frequently insert “A” over “C” opposite OG while repair polymerases (β/λ) exhibit a higher tendency to incorporate “C” over “A” opposite OG.^18^

Oxidized nucleobases are among the most common and well-studied DNA lesions, and ca. 200,000 oxidatively damaged DNA bases are produced per cell per day.^11^ Guanine is the most vulnerable nucleobase toward oxidation; indeed, the most prevalent and well-studied “OG” (original gangster) of oxidized base lesions is 8-oxo-7,8-dihydroguanine (OG).^12^ The mutagenic potential of OG arises from its ability to base pair like the canonical base thymine (T), leading to adenine (A) misincorporation during replication forming an OG:A mispair; subsequent replication of the mispair seals the G:C to T:A transversion mutation (Figure 1).^8,13^ Increased mutagenesis due to the accumulation of OG in the genome provided the basis for the free radical theory of aging^14^ and is correlated with a variety of diseases, including neurodegenerative disorders and various types of cancer.^8,15,16^ Most relevant to this review, the accumulation of OG in the tumor suppressor gene *APC* due to inherited defects in OG repair has been linked to the formation of colorectal polyps and a predisposition to colorectal cancer.^17^

The mutagenic consequences of OG accumulation in the genome make it imperative for cells to devote the means to respond appropriately.^9^ Lesions such as OG that represent subtle chemical nucleobase modifications are typically removed by the base excision repair (BER) pathway, where lesion-specific glycosylases hydrolyze the *N*-glycosidic bond between the base and sugar, leading to the formation of an apurinic/apyrimidinic (AP) site.^9,19^ Downstream repair enzymes, such as AP endonucleases, polymerases, and ligases, excise the AP site, restore the proper nucleotide, and seal the nicked duplex to complete the process of repair. The bacterial glycosylase MutM (also known as Fpg) and the human glycosylase hOGG1 are responsible for the removal of OG bases from OG:C base pairs (bps). However, pro-mutagenic OG:A mispairs are substrates of the MutY and MUTYH glycosylase in bacteria and humans, respectively. MutY enzymes are unusual among BER enzymes in catalyzing the removal of an undamaged but inappropriately placed adenine base from OG:A rather than the damaged OG base itself; however, this process provides the crucial last stand against the mutation.^9,20,21^ Bacterial genetic studies have shown that *mutY*^*–*^*mutM*^*–*^*E. coli* has extremely high mutation frequencies relative to single-deletion *mutY*^*–*^ or *mutM*^*–*^*E. coli* strains, indicating synergy in the activity of the two enzymes toward the same lesion.^13,21^ MutY together with MutM comprises what is termed the “GO” repair pathway to prevent mutations associated with OG (Figure 1).^21,22^ The importance of preventing OG-associated mutations to maintain genomic integrity is also reflected in the high level of conservation of the GO-repair pathway across all domains of life.^23,24^

BER glycosylases took center stage upon discovery of the link between inherited variants in MUTYH and colorectal polyposis and cancer; indeed this represented the first example connecting inherited BER glycosylase defects and human disease.^17^ The recessive inherited disorder is now referred to as MUTYH-associated polyposis, or MAP, and is characterized by the accumulation of somatic G:C to T:A mutations in the tumor suppressor *APC*, leading to the polyposis phenotype and high penetrant colorectal cancer.^16,17^ The David laboratory played a key role in the initial discovery of MAP by discerning the functional impact of the two founding variants, Y179C and G396D, using the bacterial enzyme as a model.^17,25^ Notably, *in vitro* assays revealed that both bacterial variants exhibit defects in the recognition and excision of OG:A mismatches.^25,26^ Since the original landmark study, we have found that the mouse and human enzymes behave similarly.^27,28^ Of note, we consistently observed greater dysfunction for the Y179C MAP variant in *in vitro* assays than G396D, and this may be clinically significant.^16^ A plethora of other MUTYH variants, including a multitude of missense mutations, have since been associated with MAP and other cancers. Many MUTYH variants exhibit adenine glycosylase activity similar to the WT which presents a challenge for assessing the potential impact in people. Indeed, these findings prompted us to develop cellular assays to determine the impact in cells and reveal in molecular detail the intricacies of the various steps in the overall process of repair mediated by MutY and MUTYH. We have summarized our contributions to the discovery of MAP and much of our work on MutY and MUTYH variants in several reviews that we direct the interested reader to consult for additional details.^8,16,20^

Our aim in this review is to provide an overview of the research that our laboratory has performed to understand the molecular origins of the exquisite specificity and efficiency with which MutY enzymes act on OG:A mispairs within DNA. Indeed, the rarity of OG:A mispairs and their similarity to T:A base pairs (bp) make the task of MutY both onerous and remarkable. The fidelity of MutY-mediated repair requires the verification of both OG and A to allow for contextually appropriate repair to avoid removing A from T:A bps, preventing genomic mayhem by the random generation of abasic sites. Our laboratory, in concert with our collaborators, has used an array of chemical biology approaches leveraging nucleic acid chemistry, *in vitro* ensemble and single-molecule assays, structural studies, and cellular assays to provide molecular insights into the many facets of this remarkable enzyme.

## MutY Is a Retaining Glycosylase Featuring a Covalent Intermediate

**Figure 2:**
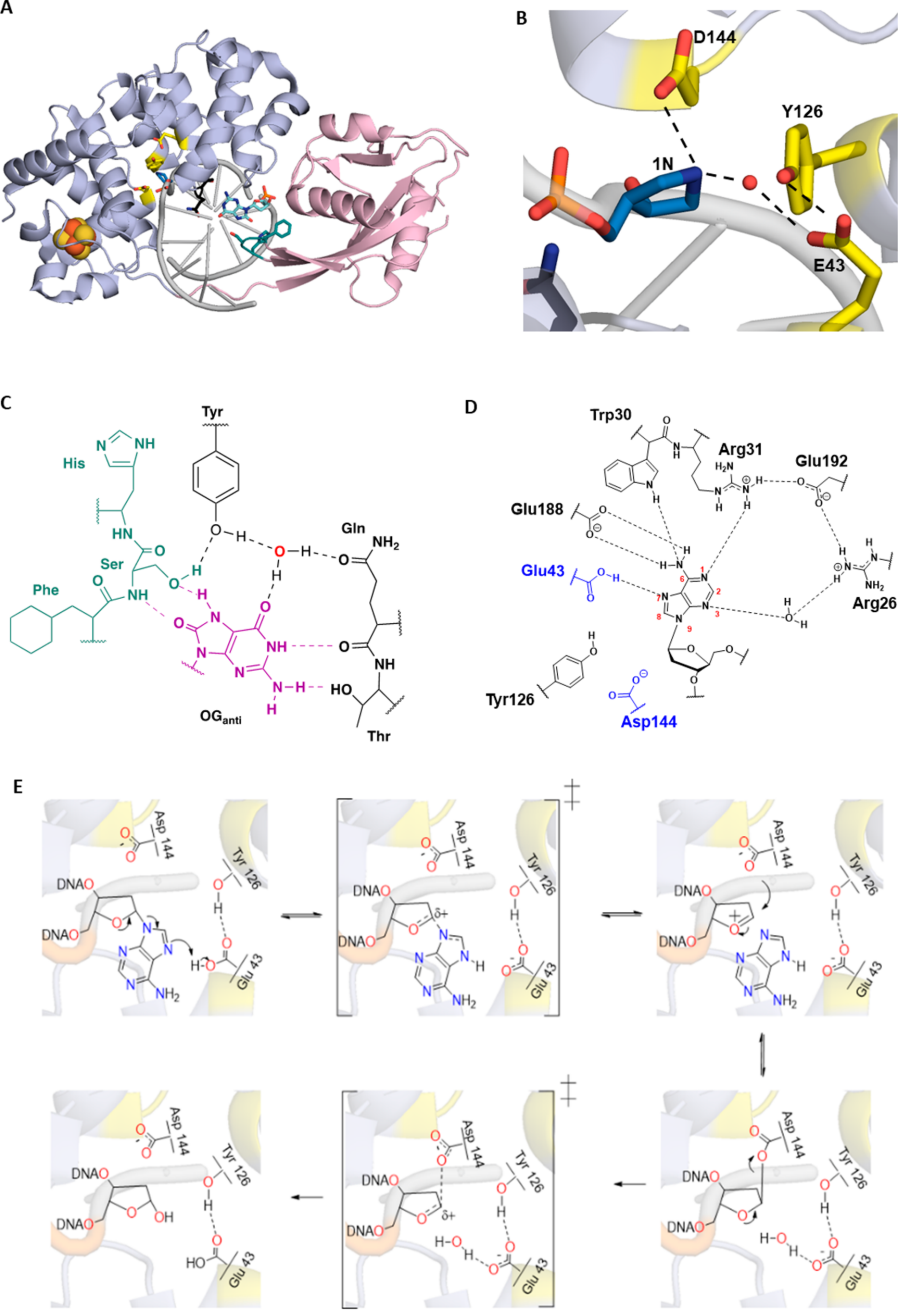
Crystal structures of *Gs* MutY reveal details of the catalytic mechanism. (A) Overall structure of *Gs* MutY bound to DNA containing transition-state analog 1N across OG (PDB ID 6U7T). (B) Extruded 1N in the active site and active site residues (PDB ID 6U7T). (C) Key residues from the C- and N-terminal domain form a network of interactions around OG_anti_. (D) Contacts observed in FLRC (PDB ID 3G0Q)^34^ to the A base flipped into the active site within the N-terminal domain. (E) Proposed mechanism for MutY-mediated adenine excision. Protonation of the A at N7 enables it to leave as a neutral molecule. The oxacarbenium ion hence formed is stabilized by the formation of a transient acetal covalent intermediate with the catalytic aspartate; subsequent hydrolysis of the acetal intermediate leads to the formation of the abasic site product. Panel E was adapted with permission from ref (38).

MutY enzymes catalyze the removal of adenine by the hydrolysis of the *N*-glycosidic linkage of 2′-deoxyadenosine with expected mechanistic similarities to the acid-catalyzed depurination of 2′-deoxy-adeosine in nucleosides and DNA.^29^ Kinetic isotope effect studies by McCann and Berti provided evidence that adenine excision by MutY follows an S_N_1 reaction mechanism utilizing N7 protonation and general acid catalysis.^30^ Structural studies, site-directed mutagenesis, and pH-dependent adenine glycosylase assays have revealed the structural domains and key amino acids involved in MutY adenine excision catalysis (Figure 2).^31,32^ MutY is a member of the helix-hairpin-helix (HhH) superfamily of BER glycosylases that “flip out” the target base into a catalytic pocket for excision.^24^ A defining feature of the HhH BER superfamily is the presence of a critical catalytic Asp that is within a Gly-Pro-rich region, referred to as a GPD motif. The Asp in MutY enzymes is required for catalysis, and pH dependence and site-directed mutagenesis have shown that maximal activity requires a deprotonated Asp.^32^ The Asp was initially proposed to be involved in activating the water nucleophile, as suggested for other BER glycosylases;^33,34^ however, a structure of *Geobacillus stearothermophilus* (*Gs*) MutY bound to DNA containing a pyrrolidine transition state (TS) mimic (1N) paired with OG (referred to as TSAC, for transition-state analog complex) inspired us to propose an alternative mechanism (Figure 2). Indeed, the close positioning of the Asp to the N1′ of 1N, which corresponds to C1′ of the target A, and the presence of a potential nucleophilic water molecule on the opposite face of 1N in the position of the departed adenine (Figure 2B) suggested that the Asp (144 in *Gs* MutY) forms a covalent acetal DNA intermediate, thereby stabilizing the oxacarbenium ion while adenine departs as a neutral leaving group.^31^ The inference from the TSAC structure suggested that the stereochemistry at C1’ would be retained, and this was confirmed by two-dimensional NMR stereochemical analysis of the acetal product formed via methanolysis in MutY reactions containing methanol in the buffer. Notably, in this mechanism, Glu43 plays dual roles as the general acid in protonating the adenine base to enhance its departure and as the general base in deprotonating the water molecule to activate it as the nucleophile. The TSAC structure also suggested roles for Tyr126 in electrostatic stabilization of the oxacarbenium ion TS and for Glu 43 in positioning the water molecule for hydrolysis of the covalent acetal intermediate.^31^

Recent work by our laboratory combining mutation of an Asn residue that H-bonds to the catalytic Asp with an alternative substrate, purine (P), paired with OG, and crystallization under different conditions led to a suite of new *Gs* MutY complex structures with the substrate and three enzyme-generated products.^35^ In all three product complex structures, only the *beta*-anomer was observed, consistent with retention of configuration and aligning with the mechanism we had previously proposed. In addition, two recent computational studies have provided additional support for this mechanism that is unique to MutY relative to other glycosylases.^36,37^

## Structure–Activity Relationships (SAR) for OG:A Repair by MutY

**Figure 3:**
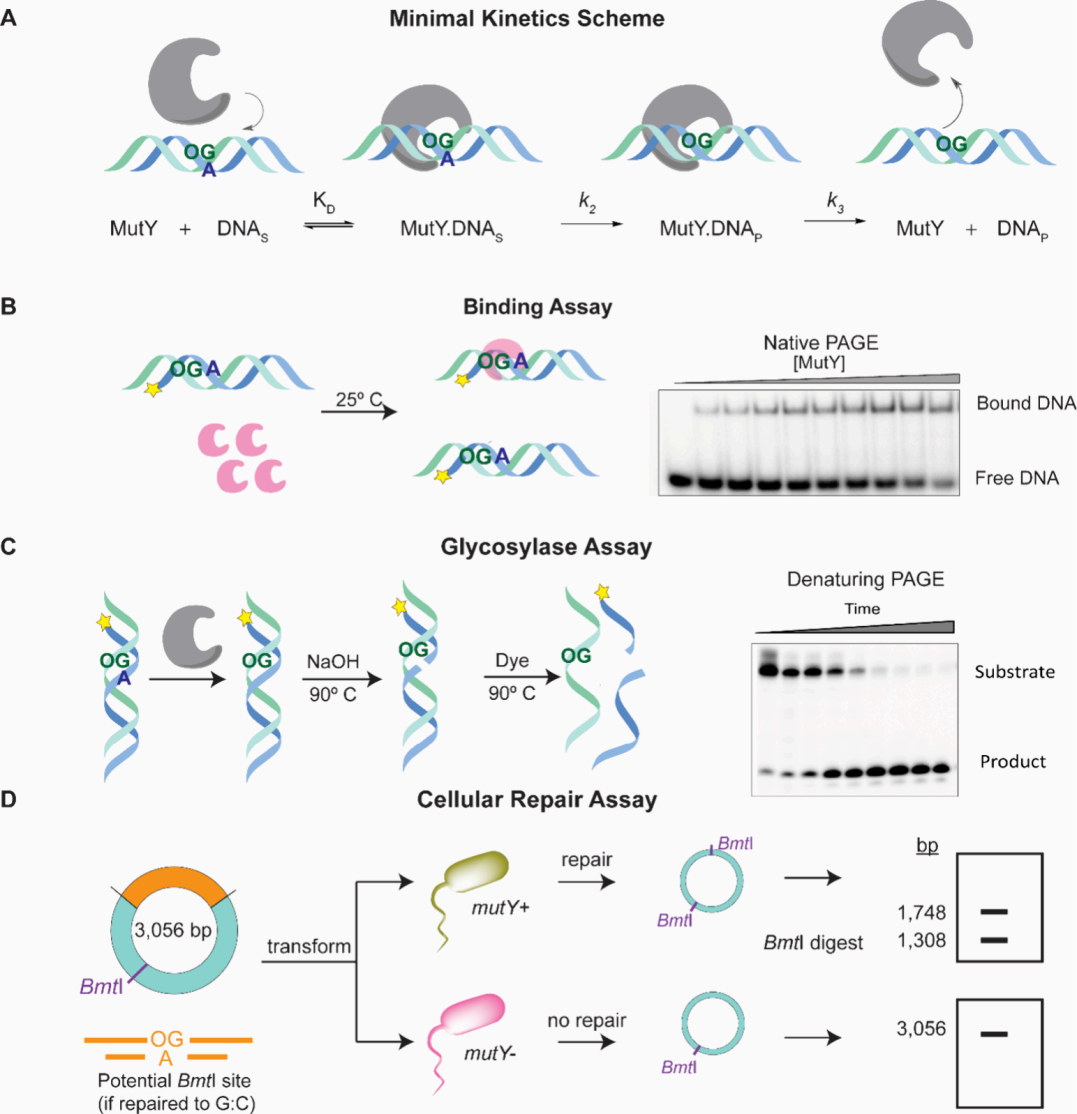
Schematic description of assays used to evaluate different stages of repair. (A) Minimal kinetics scheme depicting the major steps in the enzymatic activity of MutY in terms of the binding constant, *K*_D_, rate of adenine excision, *k*_2_, and rate of abasic site product release, *k*_3_. (B) Electrophoretic mobility shift assays, using either a catalytically inactive enzyme or a nonhydrolyzable substrate, are employed to evaluate the binding affinity of the enzyme for substrate analogs. (C) Glycosylase assay used to evaluate kinetic parameters *k*_2_ and *k*_3_. (D) *E. coli*-based cellular repair assay used to evaluate the overall extent of repair in terms of the conversion of an OG:A mispair to G:C. An in-depth description of biochemical and cellular assay methods can be found in refs (44), (45), and (46).

**Figure 4:**
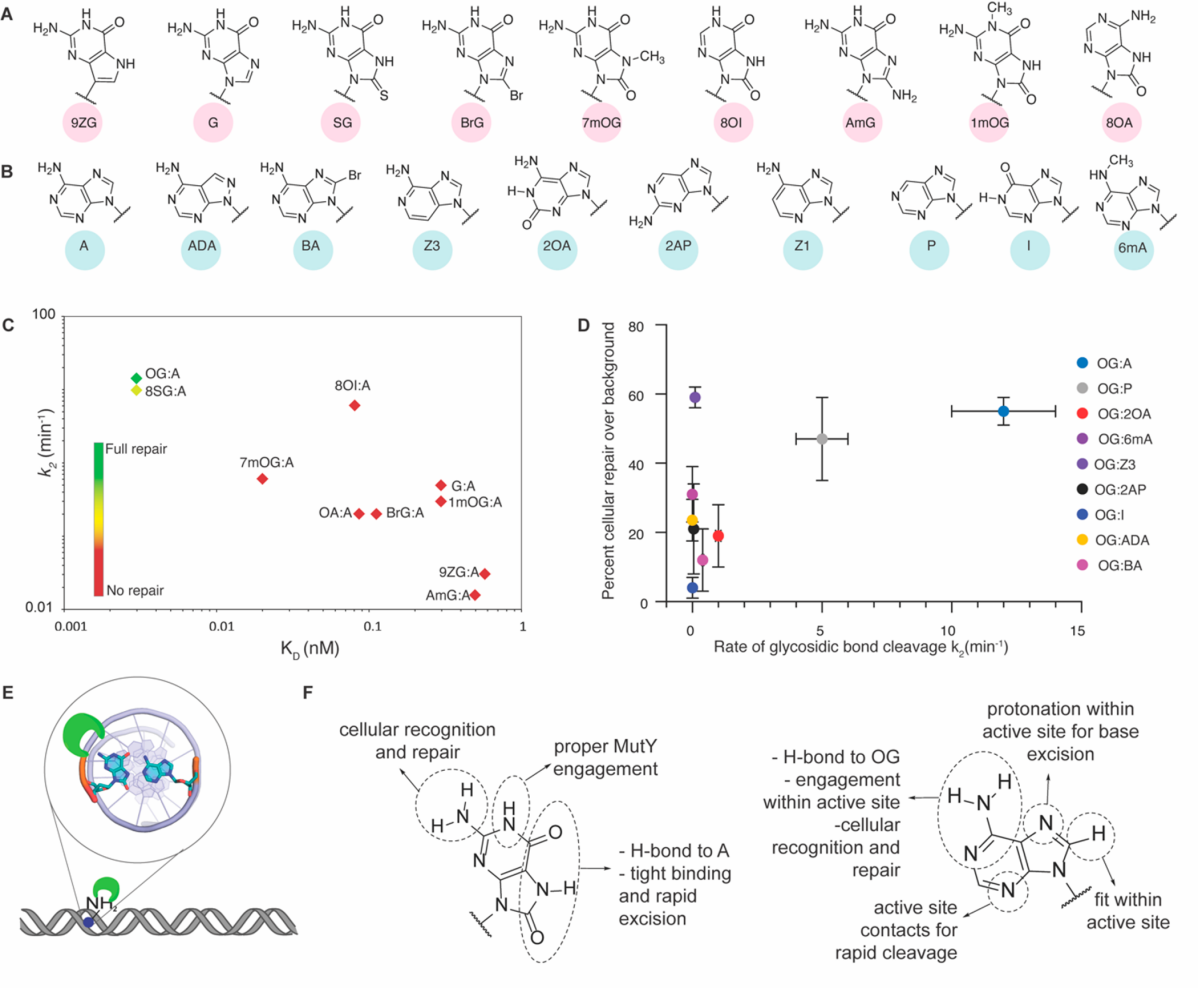
Structure activity relationships (SAR) studies reveal key features needed for recognition and repair by MutY. (A) Chemical structures of OG analogs. (B) Chemical structures of adenine analogs. (C) Relationship between the rate of A cleavage and binding constants of the catalytically inactive E37S MutY variant to duplexes containing X:A mispairs, where X is the OG analog. (D) Relationship between the rate of excision of adenine analogs and overall cellular repair in *E. coli*. The adenine analogs exhibited tight binding (*K*_D_ < 5 pM) when paired opposite OG. (E) The unique H-bonding ability of A to OG positions the 2-amino group in the major groove of DNA to enable recognition by MutY. (F) Summary of the roles of structural features of OG and A as determined by SAR analysis. Adapted with permission from refs (1) and (2), copyrights 2017 and 2020, respectively, American Chemical Society.

Inspired by the medicinal chemistry approach of defining structure–activity relationships (SAR) of small molecules and natural products to gain insight into their biological targets, we have used analogs of OG and A to develop SAR for MutY.^2,39,40,41^ Structures analyzed in the SAR studies included the structure of *Gs* MutY bound to a noncleavable A analog, 2′-deoxy-2′-fluoroadenosine opposite OG, which is referred to as the “fluorine lesion recognition complex” or FLRC. In the FLRC, the A is extruded from the helix and placed in the catalytic pocket within the N-terminal domain, and the OG base has been rearranged to its *anti* conformer remaining stacked within the DNA helix with contacts from both C- and N-terminal domains (Figure 2). Since initial lesion detection and interrogation by MutY occur when the target bp is within the DNA helix, we also considered the structure of an OG_*syn*_:A_*anti*_ bp within duplex DNA in our SAR studies (Figure 1). In terms of activity (Figure 3), we analyzed the glycosylase activity of MutY on the modified substrates using a minimal kinetic scheme to define the core parameters of DNA duplex affinity (*K*_d_), base excision catalysis (*k*_2_), and DNA product release (*k*_3_).^42^ The kinetic parameters *k*_2_ and *k*_3_ were measured using gel-based glycosylase assays that monitor strand scission at the A or A analog nucleotide and were used under conditions of single or multiple turnover conditions to isolate the relevant rate constants. To simplify binding affinity measurements, we used catalytically inactive E37S MutY that binds with high affinity to substrate OG:A bp-containing duplexes but is unable to mediate base excision.^43^ To evaluate the MutY activity in a cellular context, we developed a repair assay that uses a plasmid carrying a site-specific OG:A, OG:X, or Y:A mispair strategically positioned within a BMT1 restriction site such that repair of G:C will restore the restriction site.^44^ After transformation of the lesion-containing plasmid into *muty*^*+*^ or *muty*^*–*^*E. coli* cells, amplification and extraction, restriction digestion analysis, and DNA sequencing were performed to determine the distribution of bps at the lesion site (Figure 3). In these assays, OG:A lesion bps within the plasmid DNA are fully repaired in the presence of MutY to the correct G:C bps (>95%), while in the absence of MutY a mixture of G:C and T:A bps is observed at the location of the lesion site (35% G:C, 65% T:A), consistent with the equal replication of both bp partners and the expected levels of correct versus mutagenic replication opposite OG. The extent of MutY-mediated repair of the OG or A analogs is determined by comparing the differences in *muty*^+^ versus *muty*^–^ cells. Using this multipronged approach with a series of OG and A analogs (Figure 4), we revealed exquisite detail on the features important for repair of OG:A bps by MutY.^2,41^

The selected OG and A analogs systematically modified different structural aspects of the target mispair and revealed details of specific aspects of the repair process mediated by MutY. Indeed, these studies highlighted the importance of *OG* detection in the search and rescue mission of MutY. Alterations to the OG structure decreased both the binding affinity to MutY and the catalytic rate of A excision, indicating the high degree with which the structure of OG is required for proper engagement in the OG binding site. Indeed, OG analogs retaining the 8-oxo (or chemically analogous 8-thio) structural feature exhibited the highest affinity for MutY, indicating the importance of the thymine-like Hoogsteen face of OG for high affinity to MutY. In contrast, adenine analogs retained high affinity for MutY and were excised to near completion as long as they were paired across OG,^2^ suggesting sequential recognition of the base-pairing partners, with the OG structure guiding initial recognition.

**Figure 5:**
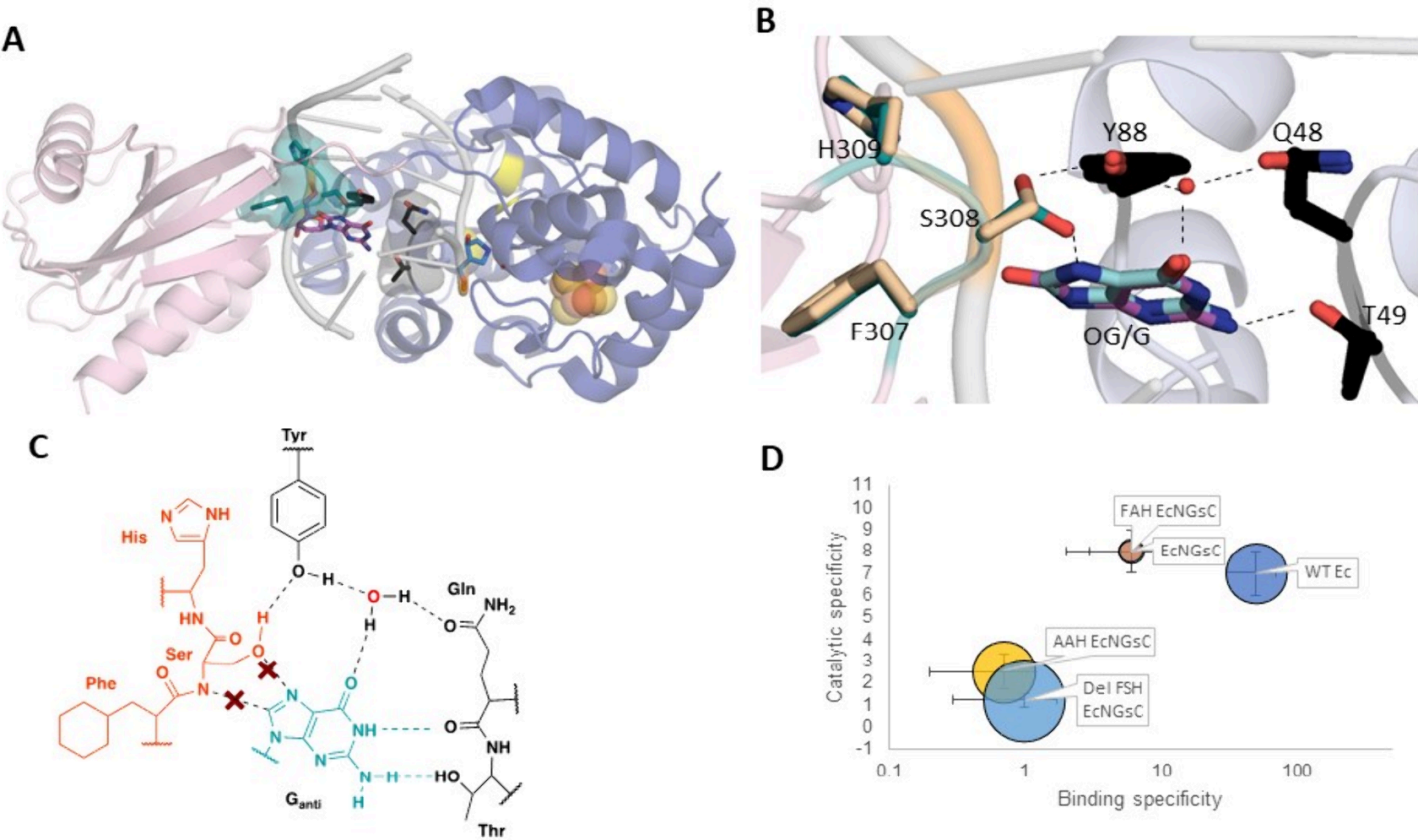
Recognition of OG:A by MutY is dependent on a conserved C-terminal loop. (A) FSH loop invades the DNA helix searching for OG (green, PDB ID: 6U7T). (B) Overlay of crystal structures of *Gs* MutY bound to the transition-state analog paired opposite OG (magenta; PDB ID: 6U7T) and G (cyan; PDB ID 6Q0C) showing the invasion of the DNA helix by the FSH loop. The FSH loop serine (S308 in *Gs* MutY) is proposed to aid discrimination between OG and G through the formation of an OG-specific hydrogen bond. (C) Key residues from C- and N-terminal domains form a network of interactions around OG_anti_ while G_anti_ lacks the interactions (denoted with red X) with the serine residue that has rotated 120° but maintained interactions with tyrosine. (D) Graph showing the binding and catalytic specificity of FSH loop mutants. The mutation suppression frequency is represented by the size of the circles corresponding to each variant. Smaller circles indicate that the MutY variant is competent in suppressing G:C → T:A mutations in cells.^3^ Data in panel D was taken from ref (3).

The high sensitivity of the adenine excision rate constants *k*_2_ to OG structural modifications was somewhat surprising due to the distal location of the OG binding pocket from the active site. In addition, the impact of OG modifications on MutY glycosylase activity did not correlate with altered duplex stability, indicating that altered base-pair disruption and base flipping alone are not the origins of MutY’s high sensitivity to structural deviations from OG.^1,47^ The long-range impacts suggest that OG:A lesion disruption and engagement elicits a conformational change that “locks” MutY into a catalytically competent state that is enabled only by the chemical structure of OG.^1^ As shown in the FLRC structures, after localizing to the mispair, MutY rotates OG from its *syn* conformation when in the duplex to an *anti* conformation while also extruding the A nucleotide out of the helix to place the adenine base into an adenine binding pocket. Once lodged in the adenine binding site, extensive H-bonding contacts to every heteroatom on A are made to align the A nucleotide for contacts with the catalytic Glu and Asp residues (Figure 5C).^34^ Correlation of experimental acid labilities and gas-phase acidities with *in vitro* excision rates of A analogues revealed that the H-bonding patterns also modulate the acidity of the A to affect its rapid protonation and release. These analog studies demonstrate that only adenine was rapidly oriented and cleaved within the active site of MutY.^2^

The effect of OG and A structural modifications on MutY-mediated repair in *E. coli* revealed how differences observed *in vitro* translate to a cellular context. These results drew even more attention to the high reliance on the recognition of unique features of OG by MutY for facile repair. Despite exhibiting a range of reduced activity *in vitro*, all of the OG analogs tested except 8-thioG, which is the most similar to OG, exhibited minimal repair in the bacterial cell assay. Most surprising to us was the observation of minimal repair of 8OI:A bps since these were found to be good substrates *in vitro*. There are contacts made to the 2-amino group of OG in the FLRC that might account for some reduction in the lesion affinity and glycosylase activity; however, it was not immediately obvious why the loss of 2-amino would be so much more dramatic in cells. An inspection of the structure of the OG_*syn*_:A_*anti*_ bp within the DNA helix provided the potential rationale: the 2-amino group of OG is uniquely located in the major groove of DNA in OG_*syn*_:A_*anti*_ bps. Indeed, in G:C and OG:C bps, the 2-amino group is located in the minor groove. In addition, the major groove methyl group of T in T:A bps is distinctly different from a 2-amino group. In the case of the A analogs, MutY-mediated repair was independent of **in vitro** rate of A excision; indeed, some analogs that were very poorly processed *in vitro* (e.g., 3-deazaA:OG, 200-fold slower) were repaired almost as well as OG:A while some that were decent substrates *in vitro* (G:A) were barely repaired in the cellular context. The only common feature of A analog bps that correlated with high repair was the ability to bp with the *syn* conformer of OG! In this way, the A or A analog effectively positions the 2-amino group to project from the DNA major groove. These results illustrate the indirect nature of detection of misplaced A opposite OG by relying on the unique base-pairing structure of A with OG. The disparity between processing-modified substrates *in vitro* and in cells demonstrates that in the presence of vast tracts of undamaged DNA within the genome, lesion detection and recognition rather than the rate of excision are the limiting factors for repair.^2^

## Identification of OG Sensing Residues in MutY

### Looking for Signs: The CTD of MutY Recognizes OG

MutY enzymes are distinct from other BER superfamily glycosylases in harboring a MutT-like C-terminal domain (CTD) that recognizes OG.^39,48,49^ When the CTD is removed, the *Eschericia coli* (*Ec*) MutY N-terminal domain (MutY CΔ225) was able to process both OG:A and G:A bps but with a diminished preference for the cognate OG:A lesion. Though *in vitro* catalytic activity was retained in the absence of the CTD, *E. coli* expressing MutY CΔ225 exhibited a significantly higher mutation frequency (500-fold increase)^39^ and was unable to repair OG:A in the bacterial lesion reporter assays.^32^ Both studies point to the key role of the CTD and OG recognition in the repair of OG:A mismatches in a cellular context. Since the base excised by MutY is the undamaged A paired opposite OG rather than the damaged base itself, initial recognition of the lesion is necessary to avoid indiscriminate excision of other undamaged DNA bases. The two domains of MutY enable complete engagement and interrogation of both DNA strands (Figure 2A), in contrast to other HhH glycosylases that primarily interact with the lesion-containing strand.^50^ The dual domain structure enhances substrate specificity as observed by the fact that MutY can recognize two structurally related DNA lesions, OG:A and G:A. However, subtle differences in structure have a pronounced effect on catalysis: MutY processes G:A almost 30-fold less efficiently than OG:A *in vitro* and negligibly repairs G:A in a cellular context.^41,44^ From these findings, we suggest that the *in vitro* excision of A from G:A mismatches by MutY is a precarious evolutionary artifact rather than a biologically relevant activity, especially since the mismatch is corrected by the mismatch repair pathway.^51^

### Structural Studies Reveal the Importance of an “FSH” Loop in Damage Detection

To reveal insight into motifs in MutY responsible for OG specific recognition, we swapped OG for G and determined a structure of *Gs* MutY bound to the pyrrolidine transition-state analog, 1N, opposite G (TSAC-G:1N), in collaborative studies with the Horvath laboratory (University of Utah).^3^ The structural overlay of the TSAC-G:1N structure with the corresponding OG:1N structure (TSAC-OG:1N PDB ID 6U7T) showed remarkably similar structures overall with the only changes localized at Ser308 within the CTD. The perturbed Ser308 normally H-bonds to the N7–H of OG but rotates away and disengages from the N7 lone pair of G (Figure 5B). The Ser308 rotamers in both structures retain H-bonding to Tyr88 (Tyr179 in human MUTYH, founder MAP variant) and preserve the connection of N7–H and O8 of OG with Tyr88 *Gs* MutY. Tyr88 also forms a hydrogen bond with a water molecule that also interacts with Gln48 and O4 of OG (Figure 5C). The Watson–Crick face of OG forms hydrogen bonds with two other N-terminal domain residues, Gln48 and Thr49, that intercalate into the void left by the extruded adenine (Figure 5C). Conspicuously, Ser308 resides at the tip of a β-hairpin loop that penetrates the DNA helix and positions itself close to OG (Figure 5B). The network of interactions mediated by Ser308 and the presence of a similar loop in the d(OG)TPase, MutT, implicated the FSH loop as an essential OG recognition motif (Figure 5).

The role of the FSH loop in ensuring OG:A repair in a cellular context was further demonstrated using a rifampicin resistance assay^46,52,53^ to measure mutation suppression by MutY variants in which the amino acid side chains were replaced or the loop was deleted entirely (Figure 5). The toxicity of full-length *Gs* MutY expression in the reporter *E. coli* strain necessitated using a chimera containing the N-terminal domain of *E. coli* MutY and C-terminal domain of *Gs* MutY (*Ec*N*Gs*C MutY). We observed that single amino acid replacements in the FSH loop minimally impacted the mutation frequency, whereas double amino acid substitutions or loop deletion resulted in significant increases in mutation frequencies, showing that the residues cumulatively play a role in OG:A repair (Figure 5).^3^

*In vitro* binding and kinetics experiments additionally demonstrate the importance of the FSH loop in the discrimination of OG from undamaged G. For this discussion, we define the “binding specificity” as *K*_D_ of MutY for G over OG and “catalytic specificity” as *k*_2_ for OG over G. Single substitution of Ser308 to an alanine (FAH *Ec*N*Gs*C) decreased the affinity for both OG:FA and G:FA duplexes while maintaining binding specificity. The double replacement AAH variant and loop deletion (Del FSH) impacted binding more drastically, indicating that both residues are necessary for binding and catalytic specificity for OG (Figure 5).^3^

## Seeing Is Believing: Single-Molecule Studies Reveal the Key Role of His in the FSH Loop of MutY to Detect OG:A bps

Accurate and effective selection of damaged bases by glycosylases within a vast excess of undamaged DNA is a statistically daunting feat that for decades has fascinated the DNA repair community.^54^ Many BER glycosylases are able to identify subtle chemical and/or structural modifications that are minimally disruptive or destabilizing to the DNA helix, thus providing no obvious signposts to telegraph their location. It has been postulated that glycosylases utilize facilitated diffusion to slide along the DNA backbone to detect minor alterations in DNA structure which is achieved through thermal energy alone, rather than ATP hydrolysis as in the case of DNA helicases, MutS, and other DNA repair enzymes.^55,56^

Direct visualization of glycosylase motion by single-molecule fluorescence microscopy (SMFM) has provided invaluable insight into glycosylase motion.^57−60^ By stretching λ-DNA across silica beads to create DNA tightropes, trajectories of single Q-dot-labeled glycosylases have been visualized in real time.^58,61,62^ Using this approach, the glycosylase hOGG1 was shown to repeatedly interrogate a section of DNA through repeated one-dimensional scanning.^62,63^ Despite the redundancy in this search mechanism, the probability of OG capture is enhanced by increasing the number of encounters between glycosylase and the lesion.^63^ Subsequent studies with *Ec* glycosylases Fpg, Nei, and Nth showed similar diffusive behavior wherein each glycosylase repeatedly scanned a single area of DNA.^64^ Studies using DNA with randomly introduced abasic sites showed that glycosylases “pause” more frequently on damaged DNA.

**Figure 6:**
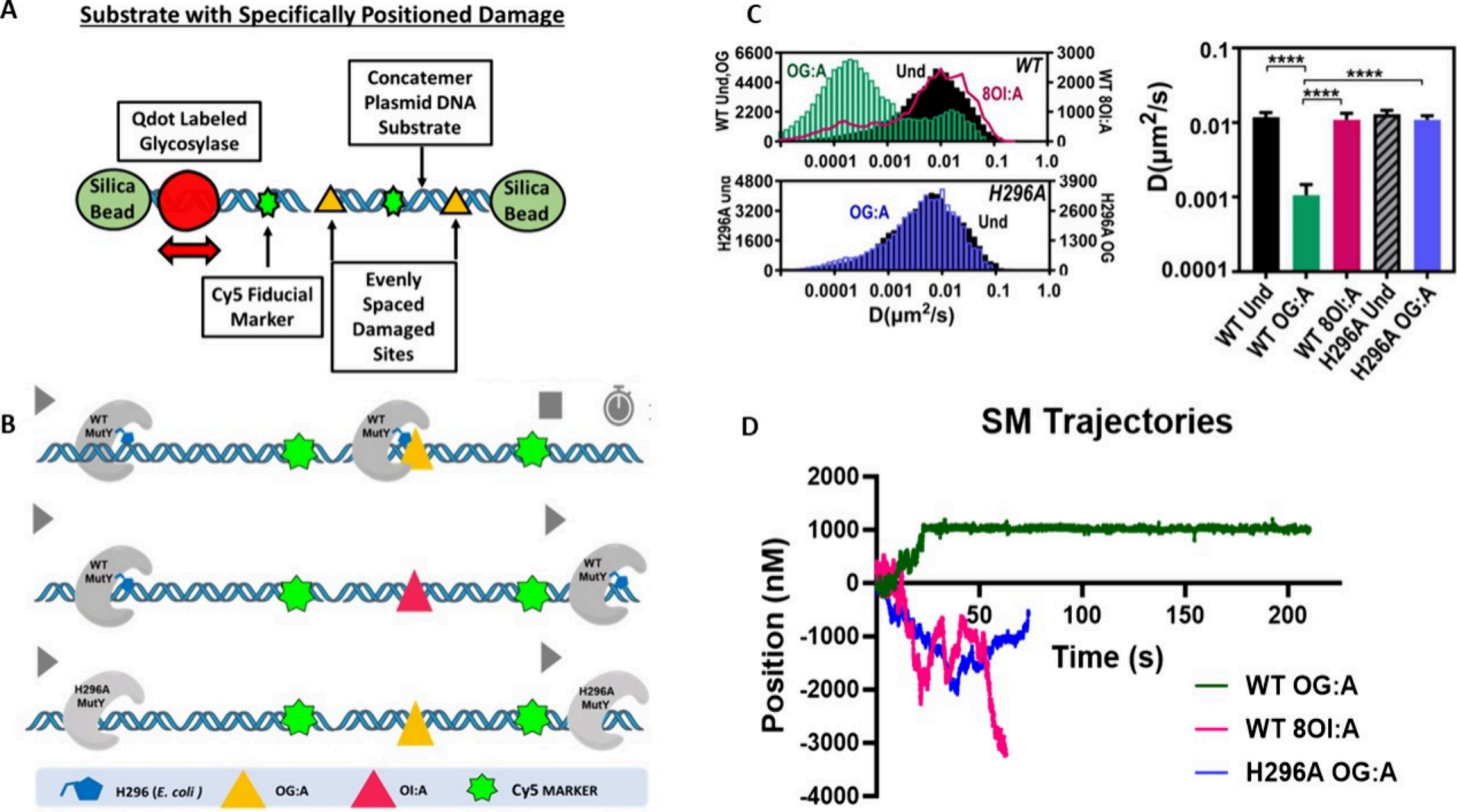
Single-molecule studies using *E. coli* MutY revealed the role of the C-terminal domain histidine, H296, in OG:A detection. (A) Overview of single-molecule-based assay utilizing Q-Dot conjugated glycosylase molecules to monitor in real time diffusion on lesion- or nonlesion-containing stretched DNA. Lesions are equally spaced between CY5 markers to correlate periods of “pausing” with the sensing of lesions. (B) Cartoon depiction highlighting observed outcomes when WT MutY is monitored in search of OGA (top) OI:A (middle) histidine 296 searching for OG:A (bottom). (C) Representative displacement trajectories of WT MutY and H296A variant on an OG/OI:A lesion containing concatemerized substrate DNA. (D) Histogram showing counts for individual glycosylase molecules and computed diffusion rates. The figure was adapted from ref (4), copyright 2020, American Chemical Society.

Single-molecule fluorescence microscopy search assays performed in collaboration with the Lee laboratory (University of Vermont) were used to directly observe the real-time search behavior of WT *Ec* MutY on OG:A and 8OI:A bps to test the importance of the 2-amino group in the lesion recognition process (Figure 6).^4^ These studies directly visualized Q-dot-labeled MutY searching DNA tightropes containing OG:A or OG:A bps positioned every 2726 bps that were identifiable via their relative positioning to a fiducial Cy5 label. SM trajectories showed long-pause events for MutY with OG:A bps (>300s) while noticeably absent were such long pauses for WT MutY on 8OI:A sites. Time-weighted sliding window diffusion analysis revealed that MutY scans rapidly on undamaged DNA tightropes at rates consistent with random diffusion (*D*_max_ ≈ 0.01 μm^2^/s) while the presence of OG:A sites leads to a significant decrease in diffusion rate consistent with pausing (*D*_max_ < 0.001 μm^2^/s). In the presence of 8OI:A bps, MutY shows primarily fast diffusion, indicating no recognition of the damaged base analog. This work demonstrates that the 2-amino group of OG is essential to the detection of the OG:A bp, and its absence leads to an inability to find 8OI:A bps and mediate their repair.^4^

The sensitivity of MutY repair to the absence of the 2-amino group in 8OI:A substrates and the conspicuous positioning of the FSH loop near OG suggested to us that a residue within the FSH loops serves as the “sensor” of interhelical OG:A bps.^4^ Modeling using several *Gs* MutY structures suggested that His 309 (*Gs* MutY) would be appropriately positioned to detect the 2-amino group of OG_*syn*_. Indeed, the *in vitro* and cellular repair of a variant at the corresponding position in *E. coli* MutY (H296A) acting on OG:A substrates mirrored the results of WT acting on 8OI:A bps. Specifically, H296A acting on OG:A and WT acting on OI:A both showed only 2-fold-reduced adenine excision (*k*_2_). In terms of binding affinity (*K*_d_), the affinity was more dramatically reduced for H296A with OG:A than WT with 8OI:A (150-fold vs ∼12-fold, respectively). Cellular repair with H296A was significantly less than WT with OG:A bps but slightly above that observed with WT on 8OI:A bps. These results suggest that in a cellular context where there is a larger amount of undamaged DNA, H296A MutY is severely compromised in detecting OG:A bps.

Single-molecule visualization of the behavior of H296A MutY on OG:A containing DNA tightropes was similar to that observed for WT MutY on undamaged DNA, indicating without the histidine, MutY is “blinded” to the lesion (Figure 6B–D).^4^ A small population of H296A MutY encountering OG:A lesions was observed; however, persistent H296A pausing at damaged sites was not observed. Only WT MutY on OG:A bps showed significantly longer binding lifetimes than the combination of H296A/OG:A or WT/8OI:A (Figure 6C,D). These results showed that the histidine residue was indispensable in initial OG:A lesion detection. Collectively, the evidence from structural studies, biochemical characterization, cellular assays, and single-molecule visualization demonstrates that the FSH loop is important for detecting and binding to OG:A. These results demonstrated that both the 2-amino group of OG and His296 of MutY are required for the detection of OG:A bps in the context of large tracts of undamaged DNA, as would be present in cells, and is borne out by the lack of repair in cells when either feature is absent.

## Implications for MUTYH, MAP, and Other Human Diseases

We developed a multipronged approach to uncovering features of lesion recognition and base excision aspects of MutY. Our work has shown successful lesion recognition results from cooperation between both domains and is dependent upon the precise base pairing pattern of OG and A. We also demonstrate the significance of a conserved CTD β-hairpin loop, bearing the residues Phe-Ser-His (FSH loop), in damage detection and OG vs G differentiation. Our SAR studies indicate that initial lesion detection is highly dependent on the OG base structure with OG and A base binding within CTD and NTD, respectively, likely occurring sequentially. The presence of OG properly lodged within the CTD exerts long-range effects on A orientation within the active site in the catalytic NTD. In addition, misplaced As are detected indirectly by their ability to hydrogen bond and form a stable bp with OG in its *syn* conformer. Once the A has been extruded, H-bonds to adenine within the MutY active site mediate proper alignment for excision. Taken together, we provide a molecular view of the damage location and excision process by MutY. In addition, this work has shown how MutY variants that are deficient in any of the steps preceding catalysis, such as lesion detection (H296A) and differential lesion binding (AAH MutY), compromise the ability of MutY to successfully repair OG:A in cellular contexts.

Our SAR results with MutY provide an important backdrop for evaluating and predicting functional and clinical consequences of MUTYH variants. Indeed, we have recently found in SAR studies of OG with MUTYH that efficient MUTYH-mediated cellular repair is also critically dependent upon the 2-amino of OG.^65^ Taken all together, our work is highly suggestive that MUTYH variants that exhibit compromised OG lesion detection and affinity will be particularly disabled in their ability to mediate repair inside cells. Our work also provides a cautionary tale that relying on a single type of assays to reveal functional properties of MUTYH variants may be exceedingly misleading and therefore underscores the need for multiple functional assays and structural studies. Among the >800 catalogued variants of MUTYH reported in clinical databases, many (∼20%) result in functional defects and are associated with cancers, including breast, ovarian, gastrointestinal, and gliomas, while most (∼70%) are variants of uncertain significance (VUS).^16,66^ These extensive and potentially elusive variants predicate the need for detailed functional characterization.

Despite its role as a tumor suppressor, MUTYH is among a host of DNA repair proteins, most notably poly(ADP-ribose) polymerase (PARP), whose inhibition presents a promising chemotherapeutic modality.^67^ MUTYH inhibition offers a broad range of therapeutic potential, as its activity is implicated in various disease phenotypes.^16^ For example, results from Gao et al. indicate that MUTYH expression is upregulated in SW780 bladder cancer cells, and shRNA knockdown of MUTYH inhibited proliferation, migration, and induced apoptosis in the cells.^68^ Additionally, in murine models of ulcerative colitis, inflammation was reduced in *Mutyh*^–/–^ mice compared to that in wild type.^69^ Moreover, MUTYH is implicated in Alzheimer’s disease (AD), a neurodegenerative disease characterized by a high degree of oxidative stress.^70−72^ In recent work, Mizuno et al. determined that MUTYH deficiency (achieved in a protease-dependent manner) reduced microgliosis and ameliorated memory impairment associated with AD by restoring hippocampal neurogenesis in mice.^72^ A comprehensive molecular mechanism detailing the contribution by MUTYH to the progression of neurodegeneration remains to be elucidated; however, studies suggest that MUTYH in neurons, under conditions of high oxidative stress, increases the accumulation of single-strand breaks that activate detrimental cell death signaling pathways.^10^ The “Dr. Jekyll” beneficial character of MUTYH in preventing mutagenesis and carcinogenesis, along with its “Mr. Hyde” dark side of causing disease, suggests the potential utility of both MUTYH activators and inhibitors. Despite the growing interest in discovering small-molecule modulators of DNA repair enzymes,^73,74^ no such molecules targeting MUTYH have been reported. As such, an understanding of the chemical basis of MUTYH-mediated repair can guide the design of DNA damage probes and inhibitors and can help relate clinically observed functional patterns to defects in the chemistry of the enzyme.
